# Nonequilibrium warm dense matter investigated with laser–plasma-based XANES down to the femtosecond

**DOI:** 10.1063/4.0000202

**Published:** 2023-09-15

**Authors:** F. Dorchies, K. Ta Phuoc, L. Lecherbourg

**Affiliations:** 1 Université, Bordeaux, CNRS, CEA, CELIA, UMR 5107, F-33400 Talence, France; 2 LOA, ENSTA, CNRS, Ecole Polytechnique, UMR 7639, F-91761 Palaiseau, France; 3 CEA, DAM, DIF, F-91297 Arpajon, France; 4 Université Paris-Saclay, CEA, LMCE, 91680 Bruyères-le-Châtel, France

## Abstract

The use of laser–plasma-based x-ray sources is discussed, with a view to carrying out time-resolved x-ray absorption spectroscopy measurements, down to the femtosecond timescale. A review of recent experiments performed by our team is presented. They concern the study of the nonequilibrium transition of metals from solid to the warm dense regime, which imposes specific constraints (the sample being destroyed after each shot). Particular attention is paid to the description of experimental devices and methodologies. Two main types of x-ray sources are compared, respectively, based on the emission of a hot plasma, and on the betatron radiation from relativistic electrons accelerated by laser.

## I. INTRODUCTION

### A. Opportunity of Tr-XANES for WDM investigation

The investigation of the so-called warm dense matter (WDM) constitutes a great theoretical, numerical, and experimental challenge,^1^ with fundamental implications in various fields of physics including micro-machining, planetology, and inertial confinement fusion.^2–7^ In this intermediate regime between condensed matter and plasma physics, the nuclei are strongly correlated without any guarantee of long-range atomic order, and the electrons are partially degenerated with non-negligible energies compared to the Fermi energy *E_F_*. The complexity of such a regime lies in the strong dynamic interplay between these two subsystems. The frontiers are not strictly defined, but the density ranges from a fraction to a few times that of the solid, the temperature is of the same order of magnitude as *E_F_* (several eVs), and the pressure can exceed the Mbar level.

Among the various techniques that have been developed to bring matter up to WDM, the use of femtosecond laser heating is rather simple, and potentially offers a unique opportunity to get deeper understanding of the electron–nuclei dynamic interplay (see the papers^8,9^ and the references therein). Indeed, the laser energy is fastly deposited in the electron subsystem, while the nuclei remain cold. Time resolved measurements make it possible to disentangle phenomena with various time scales, such as electronic transport, electron–nuclei thermal equilibration or hydrodynamic expansion. Moreover the nonequilibrium situation sheds light on the role of electrons in the lattice stability and phase transitions.^10–12^

High-performance computing numerical simulations, based on the density functional theory (DFT), are developed to calculate atomic and electronic structures and derive macroscopic properties. As *ab initio* as these methods are, they need to be constrained by experimental data. In principle, x-ray absorption near-edge spectroscopy (XANES) is a relevant measurement, since it gives access to atomic scale information, especially to the electronic structure, even in the partially disordered situations encountered in WDM. In this context, performing time-resolved XANES (Tr-XANES) measurements in samples heated by a femtosecond laser up to the WDM regime, provides valuable data to unravel the respective dynamics of the electronic structure and that of the nuclei, and thus to understand their interaction. On the other hand, such extreme conditions of matter require revisiting the “standard” interpretation (i.e., near room temperature) of XANES features.

### B. Specific requirements and laser–plasma-based Tr-XANES

A constraining specificity of WDM experiments is that the sample is locally destroyed by a single laser pulse. It is therefore mandatory to renew it shot by shot, which imposes technical constraints for most samples. This limits the available number of shots to obtain exploitable data. For this simple reason, dispersive XANES is often required. That is, the laser pulse has to be synchronized with a broadband x-ray pulse coupled to a spectrometer.

The time scales of the considered physical phenomena range from a few picosecond down to the femtosecond, requiring corresponding time resolution on the x-ray probe. Pioneering experiments have been carried out by using a synchrotron (70 ps duration) coupled with a streak camera that provided a 2 ps time resolution.^13^ A few years earlier, sub-picosecond resolution has been achieved in a pump-and-probe scheme, with the synchrotron slicing technique providing hundreds of femtosecond x-ray pulses.^14,15^ The price to pay was a greatly reduced photon flux, so that the time-resolved x-ray absorption was recorded at only a few selected energies. More recently, x-ray free electron lasers (X-FEL) have revolutionized the ultrafast x-ray science, by providing very intense femtosecond x-ray pulses. If their contribution to femtosecond x-ray diffraction (XRD) is unprecedented, their use for Tr-XANES is made challenging because of the spectral nature of the x-ray pulses: narrow band (a few eVs) with stochastic peaks due to the self-amplified spontaneous emission (SASE) mode.^16–18^

The experiments mentioned above lie on large-scale research facilities, providing necessarily limited and sometimes very competitive access. In order to overcome this limitation, a constant effort has been made to develop table-top laser-based ultrafast x-ray sources for several decades, supported by continuous progress in ultrashort lasers (peak intensity, stability, and average power). The first demonstration of a few picosecond resolution has been reported on resonant absorption line vanishing during molecule photodissociation.^19^ Then a series of ultrafast XRD experiments have been reported, taking advantage of the few hundreds of femtosecond duration of the K_*α*_ emission resulting from the acceleration of electrons in the laser–plasma interaction.^20–23^ Based on the same principle, the continuous spectrum of the bremsstrahlung emission has been considered to perform x-ray absorption spectroscopy (XAS) experiments with similar expected time resolution.^24,25^ However, the very low x-ray fluxes reported prevents its use for the study of WDM. Alternatively, the plasma thermal emission from a high atomic number target can provide broad spectra with much more photons, slightly degrading the time resolution close to the picosecond.^26^ These latter techniques lead to a mostly isotropic emission. More sophisticated laser-based x-ray sources have been recently proposed, providing both collimated beams and femtosecond time resolution. The most advanced high-harmonic generation (HHG) sources (driven by few-cycle, near-infrared laser systems) demonstrated their capabilities at photon energies up to few hundreds of eV.^27^ More recently, femtosecond Tr-XAS has been reported in the multi-keV range, benefiting from the betatron emission of a laser-accelerated relativistic electron bunch.^28,29^

Beyond the proof-of-principle, advances in ultrafast laser-based x-ray sources are now resulting in realistic and reliable Tr-XANES experiments. This is particularly the case for the investigation of nonequilibrium WDM, where strong modifications are expected in both electron and atomic structures, resulting in easily observable modifications of XANES spectra. However, these techniques can be transposed to other research fields. In this paper, we propose to illustrate such Tr-XANES development, by synthesizing a series of experiments that our team carried out on warm dense metals, with several ultrafast laser-based x-ray sources, decreasing the time resolution down to the femtosecond scale. Most of the scientific results having already been published, we pay particular attention here to the detailed description of the x-ray sources properties, the corresponding experimental setup for Tr-XANES, as well as their limits and advantages for a readership non-specialist in WDM.

## II. FEW-PICOSECOND XANES WITH SOLID TARGET X-RAY SOURCE

### A. Principle of thermal laser–plasma x-ray source with solid target

Only a few years after the discovery of the very first lasers, they were amplified at high energy to produce hot plasmas leading to intense x-ray radiation and possible nuclear reactions. The first demonstration of ultrashort laser–plasma x-ray pulse (picosecond) has been reported in the early 90s,^30^ taking advantage of the chirped pulse amplification technique.^31^

In this regime (laser duration from femtosecond to picosecond, and intensity up to ∼ 10^18^ W/cm^2^), the laser focused on a solid target generates a hot plasma at the surface, with a typical temperature ranging from 100 eV to 1 keV. Such a plasma emits naturally an intense x-ray emission, which we will call “thermal emission.” As it is not optically thick, the spectrum is not reduced to that of a black body. It is rather made up of numerous lines resulting from the complex atomic physics of hot plasmas, reaching up to a few keV. Several mechanisms at work during the duration of the laser–plasma interaction lead to the acceleration of some electrons to higher energies (up to several tens of keV).^32^ They are called “supra-thermal” or simply “hot” electrons. They generate high energy x-rays when they interact with the depth of the target, analogously to x-ray tubes, that is, by producing a few characteristic lines ( $K_{\alpha }$, etc.) on the flat spectral background of the bremsstrahlung. These hot electrons dominate the laser–plasma interaction at relativistic laser intensities ( ≥ 10^18^ W/cm^2^).

The thermal x-ray emission, as well as the one in the characteristic lines from hot electrons, is intrinsically isotropic. Indeed, these photons result from the radiative de-excitation of the plasma ions. Concerning the bremsstrahlung, the emission is preferential along the hot electron trajectory, but it is quickly isotropized by the angular diffusion. Due to the re-absorption in the target, the x-ray emission angular distribution follows the Lambert's cosine law, reducing the effective solid angle to *π* sr on the incident laser side. Optionally, the use of a thin foil target allows emission in the opposite side.

The duration of the x-rays originating from hot electrons has been observed in the hundreds of femtosecond range.^20^ It is mainly driven by the stopping power of hot electrons in the target. On the other hand, the thermal emission persists as long as the plasma is hot and dense enough to efficiently radiate. The x-ray duration is then driven by the plasma hydrodynamics (and radiative cooling). It is rather of the order of the picosecond, but it depends on the spectral range considered, as it will be discussed later.

To complete this section, one has to discuss about the x-ray conversion efficiency from the laser energy. After optimization, the hot electron population can carry about 10% of the laser energy. Then, their conversion in x-rays is driven by tabulated cross sections and depends on the electron energy and on the target material.^33^ One of the best conversion efficiency reported in molybdenum $K_{\alpha }$ (17.5 keV) reaches 10^−4^ of the laser energy after integration over *π* sr and on the natural width ( ∼6 eV).^34^ Unfortunately, such emission is monochromatic and the photon energy is fixed by the target material, which prevents any XAS measurements. The flat spectrum of bremsstrahlung is more suitable, but the corresponding level of emission is a few orders of magnitude lower. As detailed in the next section, the thermal emission of a plasma can combine a high conversion efficiency with a broad and adjustable spectrum, in the multi-keV spectral range.

### B. X-ray source characteristics

**FIG. 1.**
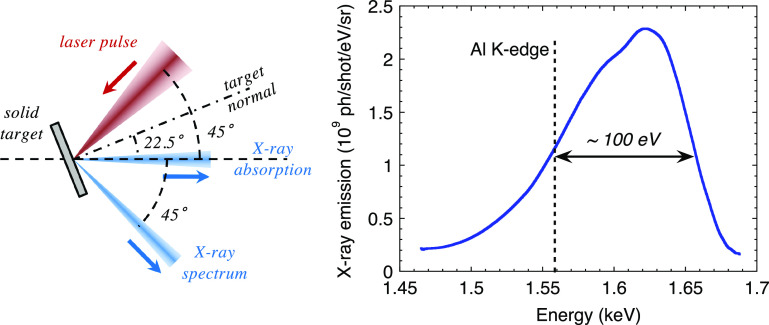
Left: scheme of an x-source generated from the laser–plasma interaction on a solid target. Reproduced with permission from Dorchies *et al.*, Phys. Rev. E **98**, 033212 (2018). Copyright 2018 American Physical Society.^36^ Right: typical spectrum of an x-ray source designed near the aluminum K-edge (100 mJ laser on a holmium target).

A typical geometry of a laser–plasma x-ray source on solid target is shown in Fig. 1 (left). The laser is focused on the target. The laser absorption, then the x-ray conversion is optimized with an incidence angle of a few tens of degrees, and P− polarization. Even if it may seem obvious, it should be noticed that the laser–plasma interaction must take place under vacuum. This is imposed by the focusing of the laser, as the associated high electro-magnetic field could ionize the surrounding air and then severely alter the laser pulse propagation before reaching the target.

As mentioned earlier, the x-ray emission is isotropic in the half space in front of the target. To illustrate the use for XAS measurements, two solid angles are just drawn. The first one (“x-ray spectrum”) is used to monitor the x-ray source spectrum (online reference). The second one (“x-ray absorption”) is sent through the sample in order to register the transmitted spectrum. The x-ray absorption spectrum is extracted from these two simultaneous measurements. Each of the detection channels may have defects (imperfect crystal reflectivity, for example). Regular calibration measurements (without the sample) can correct these sources of systematic errors.

The interest of thermal laser–plasma x-ray source is that the conversion efficiency in x-ray lines is expected to be relatively high. It is comparable to the characteristic lines, such as $K_{\alpha }$. However, the emitted spectrum is richer, since the same transition can occur on a wide range of ionization states and electron configurations. Each situation modifies the screening of the nucleus potential, thus inducing a slight spectral shift. The “K-shell” emission is composed of radiative transitions ending in the first atomic shell. It consists in separated lines. The number of lines increases in the “L-shell,” and exponentially in the “M-shell” emission. The calculation of the detailed atomic physics is then a very high and still unattainable challenge. Myriads of lines merge in large spectral structures, named unresolved transition arrays (UTA). In the particular case of ultrashort laser–plasma interaction, the combination of high density, high velocity, and nonequilibrium contributes to the lines broadening, leading to smooth and broadband features.

An example is plotted in Fig. 1 (right). A ∼100 mJ laser was focused on a holmium target ( Z= 67) in order to optimize an x-ray source near the aluminum K-edge (1.56 keV). A regular and reproducible UTA pattern was registered, with a spectral width exceeding 100 eV, suitable for XANES measurements. In this example, about $2\times 10^{9}$ photons are generated per shot, per eV and per steradian. After integration over 100 eV and *π* sr, a conversion efficiency of about 0.2% is achieved from the laser energy. For comparison, the reference^25^ reported an average emission of $2\times 10^{7}$ ph/s over 4π sr in the bremsstrahlung emission integrated from 7 to 8 keV, with a 30 mJ laser operated at 10 Hz. That corresponds to 160 ph/shot/eV/sr. Even considering that the photon energy range is higher (7–8 keV instead of ∼1.6 keV), the bremsstrahlung emission is several orders of magnitude lower, severely limiting XAS measurements to investigate nonreversible processes such as WDM.

The M-shell emission is composed of several patterns such as the one presented in Fig. 1, which are spectrally close. This allows flexibility on the choice of the conversion target, when some materials are difficult to handle. In addition, the spectral patterns slightly shift toward high energies (a few tens of eV), when increasing the laser pulse energy or its duration, as a consequence of a better coupling and the resulting higher temperature plasma.^35^ The best way to adjust the x-ray emission to a given absorption edge is to change the material of the conversion target. In the multi-keV range, the spectral difference between two neighboring elements in the periodic table is ∼100 eV. That makes it possible to continuously scan the spectral domain up to ∼4 keV. The downside is that it is difficult to predict the x-ray spectrum from a given conversion target. Even if it was possible from a physics point of view, that would require access to large-scale and possibly confidential simulation codes. For the simple purpose of x-ray source optimization, one can simply scale the spectral range with a tabulated M-edge (see Table I).

**TABLE I.** Some characteristics of laser–plasma-based x-ray sources developed on solid target for Tr-XANES experiments. The incident laser energy on the x-ray conversion target is set to 100 mJ, and its duration to 3 ps FWHM (1.3 ps rms).

XAS edge (name)	Energy (keV)	X-ray target (Z)	M1-edge (keV)	X-ray source (ph/shot/eV/sr)	X-ray on sample (ph/shot/eV)	Time resolution (ps rms)
Cu L3	0.93	CsI (55–53)	1.07–1.21	$2\times 10^{8}$	$1.2\times 10^{5}$	10.8 ± 0.2
Al K	1.56	Ho (67)	2.13	$8\times 10^{8}$	$5\times 10^{5}$	3.15 ± 0.25
Mo L3	2.52	Pb (82)	3.85	$2\times 10^{7}$	$8\times 10^{3}$	2.5 ± 0.7

For similar reasons, the x-ray pulse duration is difficult to evaluate precisely from calculations. That would require complex radiation-hydrodynamics codes. Its measurement is also a real experimental challenge. One can use x-ray streak cameras providing sub-picosecond time resolution, but it is an experiment in itself.^37^ Most of the time, the Tr-XANES experiment itself provides an upper value of the x-ray duration.

With solid conversion targets, we observed that the global emission level increased with the laser pulse duration from sub-ps to ∼10 ps (see, for example, Fig. 7 in the case of CsI target). The x-ray pulse duration was observed to increase as well (unpublished x-ray streak measurements). Therefore, in order to keep a good time resolution, a compromise is necessary. In Table I, several x-ray source data are reported when using a 3 ps laser pulse duration FWHM (full width at half maximum), corresponding to 1.3 ps rms. Each source has been developed for a specific absorption edge (first and second columns). The x-ray conversion target is indicated in the third column. The choice is sometimes constrained by practical considerations on the material handling. The corresponding tabulated M1-edge is reported in the next column. Then, the x-ray source emission level is given in the spectral range near the absorption edge under investigation. The following column gives the number of photons reaching the sample, when using the specific device described in the next section (XANES station with polycapillary x-ray optics). The last column reports the time resolution observed in Tr-XANES experiments, dominated by the x-ray pulse duration (Ref. 36 for Cu L3-edge, Ref. 26 for Al K-edge, unpublished for Mo L3-edge).

The general trend is that the x-ray emission level decreases with the targeted energy range. This is expected from the thermal emission from a plasma with an estimated temperature of several hundreds of eV. Note that near the Cu L3-edge, CsI was not the best element, but cheaper and easier to handle rather than lanthanum. At the same time, the x-ray pulse duration decreases. Indeed, an efficient x-ray emission in a higher energy range is more sensitive to the decrease in temperature during the fast plasma expansion and cooling.

### C. Experimental device for Tr-XANES measurements

**FIG. 2.**
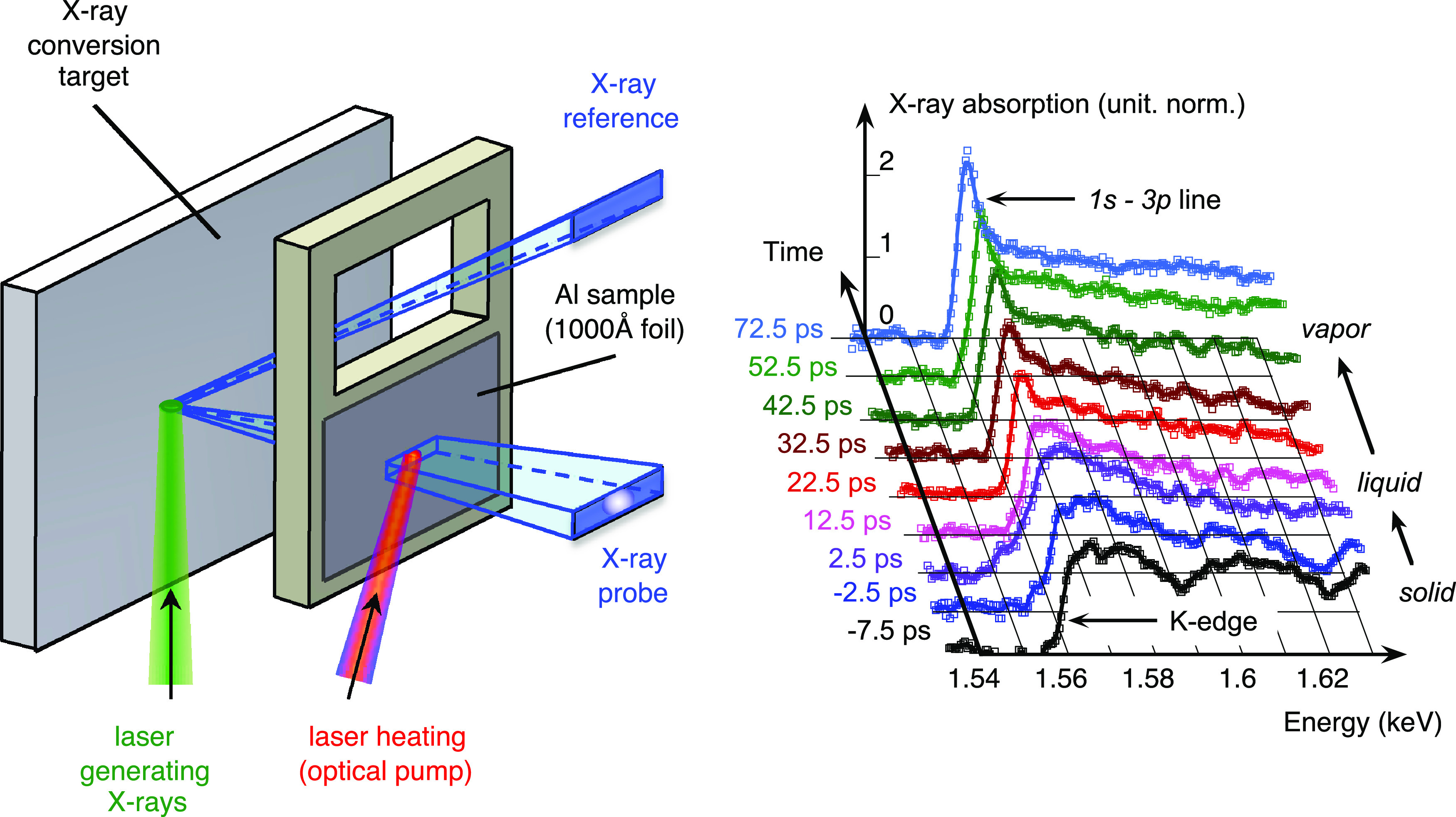
Left: scheme of the first setup used to get Tr-XANES on warm dense aluminum. The sample was set close to the x-ray conversion target. Right: some Tr-XANES spectra recorded through 100 nm thick aluminum samples, heated by a fs laser pulse at an incident fluence of 6 J/m^2^. Each spectrum results from the accumulation of ∼50 laser shots at 90 mJ and 3 ps on a holmium target). Reproduced with permission from Dorchies *et al.*, Phys. Rev. Lett. **107**, 245006 (2011). Copyright 2011 American Physical Society.^26^

The first experiments of Tr-XANES with thermal plasma x-ray source were performed near the Al K-edge, with the simple setup sketched in Fig. 2 (left). We placed the sample quite close to the x-ray conversion target (a few mm) in order to maintain a high number of x-ray photons on sample. A double crystal x-ray spectrometer was designed to simultaneously register the reference and the transmitted spectra (detailed in Ref. 38).

Some results are plotted in Fig. 2 (right). After accumulation over ∼50 laser shots, the signal-to-noise ratio was high enough on each spectrum to resolve the evolution of some features, when the aluminum turned from cold solid (negative delays) up to hot expanding plasma (longest delays).^26^ In addition to the demonstration of Tr-XANES with a table-top device, this experiment revealed original XANES features. More specifically, DFT calculations confirmed that the large modulations ( ∼30 eV period) above the K-edge, were the first oscillations of the EXAFS signal. They reflected the short-range order, which vanished faster than the 3.15 ps rms time resolution. At longer delays, the K-edge turned to the 1s−3p absorption line, signing the progressive evolution of the electron structure from the metallic conduction band of condensed aluminum (solid, then liquid) up to the atomic orbitals of expanded aluminum (vapor). In addition, the spectrum at 2.5 ps delay exhibited a broadened K-edge. That was interpreted as the specific effect of the electron temperature on the Fermi–Dirac electron distribution near the frontier between occupied and vacant states.^39^ These patterns are characteristic of the WDM regime, since they cannot be observed under more standard conditions of density or temperature.

**FIG. 3.**
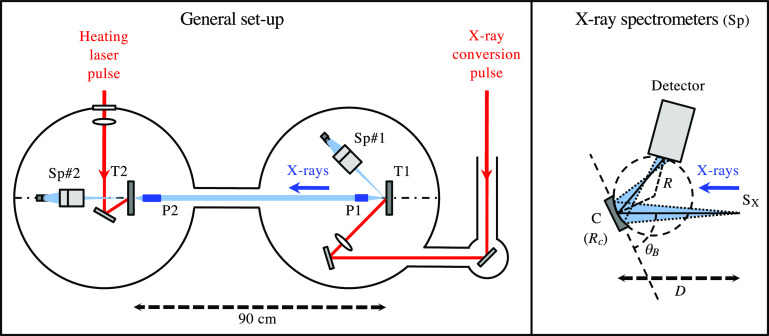
Left: sketch of the XANES experimental station developed at CELIA. X-rays are produced on the target T1, then are transported up to the sample T2 by two polycapillaries P1 and P2. Right: Detail of the x-ray dispersive spectrometers. Reproduced with permission from Dorchies *et al.*, Rev. Sci. Instrum. **86**, 073106 (2015) with the permission of AIP Publishing.^41^

Another experiment was carried out with the same setup, devoted to the ultrafast short-range disordering in warm dense aluminum.^40^ However, the overall geometry was very constrained, resulting in a complex target holder and fixed spectral range. For this reason, and to move the detector away from the plasma (which is the source of parasitic noise on the detector), we developed an experimental station dedicated for Tr-XANES measurements. It is presented in Fig. 3 and extensively detailed in the reference.^41^

The key improvement is the use of two polycapillary optics (semi-lens) in order to collect a significant solid angle of the x-ray emission, then to concentrate it onto the sample. This last is located in a vacuum chamber separated from the x-ray source. XANES spectra are extracted from the simultaneous recording of the source spectrum (reference on the spectrometer Sp#1), and the transmitted one through the sample (Sp#2). The systematic artifacts induced by the defects of the x-ray optics are corrected by the use of specific and regular calibration measurements carried out without sample. Both spectrometers are identical and can continuously scan the spectral range ∼0.5–4 keV, with rotation stages and a set of a few x-ray diffraction crystals. They are shown schematically in the right part of Fig. 3 and fully described in the reference.^41^

Each polycapillary is a matrix of hollow borosilicate capillaries, in which x-rays are reflected at grazing incidence. The non-total reflection and the losses at each polycapillary entry result in an overall transmission, which has been measured on the order of 10% from the x-ray source to the sample, over a large spectral range up to ∼10 keV. When irradiating a holmium target with a 100 mJ, 10 ps laser pulse, and considering the different filters, one gets, respectively, about $2\times 10^{9}$ ph/shot/eV/sr emitted from the x-ray source near the Al K-edge (cf. Fig. 1), then about 10^6^ ph/shot/eV on the sample, and finally ∼500 photons detected on the CCD (charge coupled device) detector per eV and per shot.

**FIG. 4.**
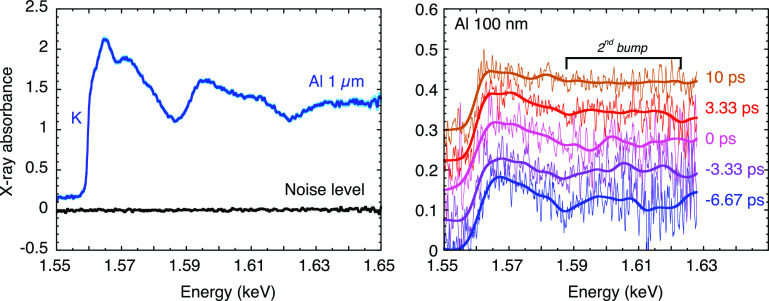
Left: K-edge XANES spectrum recorded through a 1 *μ*m thick aluminum sample, after accumulation of 133 laser shots at 100 mJ and 10 ps on a holmium target. The noise level is reported in the error bars. Right: Some Tr-XANES spectra measured through 100 nm thick aluminum samples, heated by a fs laser pulse at an incident fluence of 1.85 ± 0.25 J/cm^2^. Each spectrum results from the accumulation of 500 laser shots at 65 mJ and 3 ps on a holmium target. Reproduced with permission from Dorchies *et al.*, Rev. Sci. Instrum. **86**, 073106 (2015) with the permission of AIP Publishing.^41^

By respecting a strict experimental data acquisition protocol (detailed in Ref. 41), it is possible to overcome both the effect of shot-to-shot x-ray source fluctuation and x-ray optics defects. Finally, the noise in the extracted XANES spectra is dominated by the intrinsic uncertainty of the photon counting statistics. Depending on the required signal-to-noise ratio, several shots have to be accumulated. To illustrate that, a 1 *μ*m thick aluminum K-edge XANES spectrum is plotted in Fig. 4 (left), built after 133 shots with the x-ray source of Fig. 1. A noise level of 0.65±0.05% rms is registered around the zero absorption expected without sample.

### D. Selection of some few-ps Tr-XANES measurements on warm dense aluminum and copper

The spectrum presented in Fig. 4 (left) is quite nice. However, it is partly due to the opportunistic choice of the aluminum sample thickness. In a realistic Tr-XANES experiment, the sample thickness is not a free parameter as it must fit with the heatable depth, that is a few times the laser skin depth ( ≤100 nm in metals). Another limitation comes from the time resolution. As explained above (see Sec. II B), the laser pulse duration has to be decreased in order to guarantee a few ps x-ray pulse, thus decreasing the x-ray conversion.

Some Tr-XANES spectra near the Al K-edge are reported in Fig. 4 (right), when recorded through a 100 nm aluminum foil. The noise level in the absorbance is about 0.02 rms (i. e. 2%), but the relative noise is significant since the absolute K-edge absorbance amplitude is only ∼0.15. That being said, by accumulating over 500 shots per spectrum, and convolving over 5 eV (bold curves), it was possible to resolve the picosecond dynamics of the short-range disordering, revealed by the vanishing of the oscillations above the K-edge (first and second “bumps”).

**FIG. 5.**
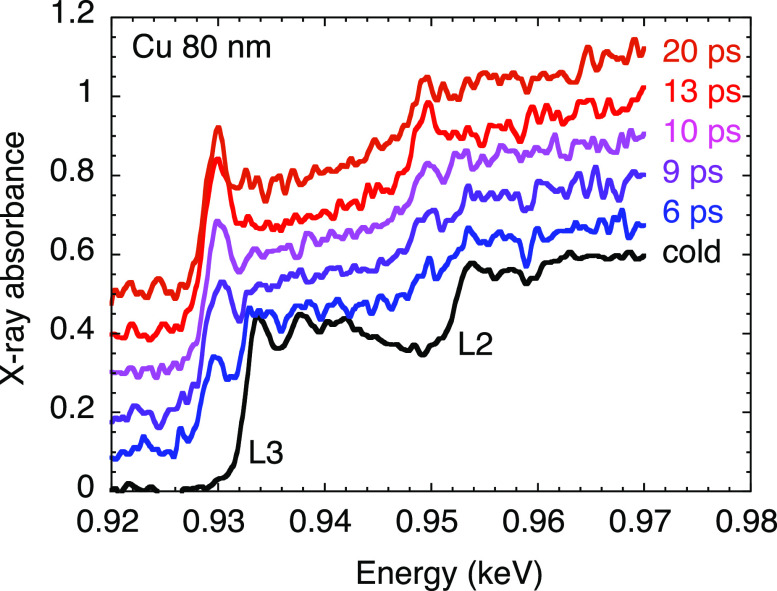
Some L3-edge Tr-XANES spectra measured through 80 nm thick copper samples, heated by a fs laser pulse at an incident fluence of 8.2 ± 1.8 J/cm^2^. Absorption spectra are artificially vertically shifted for clarity, and compared with the cold spectrum (black curves). Each spectrum is recorded after accumulation of 200 laser shots at 50 mJ on a CsI target. Error bars are not drawn so as not to overload the plot, but can be evaluated before the L3-edge. Reproduced with permission from Dorchies *et al.*, Rev. Sci. Instrum. **86**, 073106 (2015) with the permission of AIP Publishing.^41^

The study of copper L3-edge was more favorable, thanks to the combination of higher atomic number and lower energy. Figure 5 reports Tr-XANES data recorded through 80 nm thick copper foil. The amplitude of the absolute absorbance is near 0.5, and the noise level reached 0.015 (1.5%) when accumulating 200 shots per spectrum. This was sufficient to reveal the appearance and the progressive growth of a pre-edge structure, a few eV before the L3-edge.

Here again, the Tr-XANES spectra exhibit original features, which are specific to the WDM regime.^8^ Intuitive interpretation based on the electron DOS modification was confirmed by DFT calculations.^42^ The rise of the pre-edge near the copper L3-edge is the generalization of the K-edge broadening in warm dense aluminum (see Sec. II C). When the electron temperature *T_e_* exceeds ∼0.3 eV, some electrons are promoted from the d−band to higher energy states. The corresponding vacant states created in the d−band become available for photoionization, inducing a pre-edge in the XANES spectrum. In addition to this interpretation, calculations established a possible direct estimation of *T_e_*, in various thermodynamic conditions encountered in a femtosecond heating experiment (temperature up to 3 eV, thermal equilibrium achieved with ions or not, and slight hydrodynamic expansion). Further beyond the edge, some modulations have been interpreted as a signature of the crystalline structure. They will be discussed in more detail in the next section.

The rise time of *T_e_* (and therefore of the pre-edge) is expected to be as short as the laser heating of the sample, that is, in the sub-ps range. Then *T_e_* should decrease as the energy is progressively transferred to ions. The results reported in Fig. 5 indicate a poor time resolution ( ∼10 ps reported in Table I), which prevents access to this interesting physics. This motivated the development of a shorter x-ray source.

## III. PICOSECOND XANES WITH CLUSTER TARGET X-RAY SOURCE

### A. Principle of laser–plasma x-ray source with cluster target

In the 90s, cluster jets were proposed as an alternative of solid targets, combining the practical advantages of a gas jet (debris-free and continuously renewed for high repetition rate operation) with the high laser absorption of a dense medium. High x-ray conversion efficiencies were first demonstrated,^43^ before considering other applications in secondary sources of photons and particles (see the review in Ref. 44). Clusters can be produced by the partial condensation of a cooling gas in a supersonic expansion. The cluster characteristic size is nanometric, and its local density is close to the solid one. The cluster size and distribution depend on the nozzle geometry and can be monitored with the upstream pressure.^45^

**FIG. 6.**
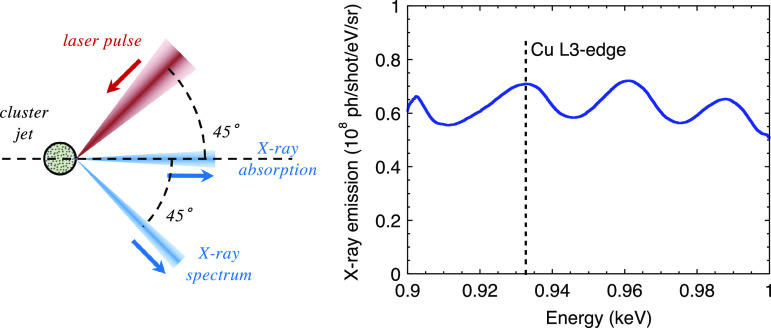
Left: scheme of an x-source generated from the laser–plasma interaction on a cluster target. Right: typical spectrum of an x-ray source designed near the copper L3-edge (100 mJ laser on a xenon cluster jet). Reproduced with permission from Dorchies *et al.*, Phys. Rev. E **98**, 033212 (2018). Copyright 2018 American Physical Society.^36^

The scheme of a laser–cluster x-ray source is shown in Fig. 6 (left). It is very similar to that of a solid target. The atomic physics that leads to the hot plasma x-ray emission is the same. The emission is isotropic and the re-absorption can even be reduced due to the average density, which is that of a gas (resulting in an emission extending over 4π sr). Figure 6 (right) reports a measurement of the x-ray emitted spectrum from a xenon cluster jet (Ref. 36). The atomic number of xenon ( Z= 54) is located in between that of cesium and iodine composing the CsI target previously used for copper L3-edge XANES studies. That results in a bright emission over the entire spectral range of interest.

The main difference lies in the laser coupling with the target. From a macroscopic point of view, a significant part of the incoming laser energy is reflected at the surface of a solid target, thus lost for the plasma heating. In a cluster jet, the laser energy scattered by a given cluster can contribute to the heating of those surrounding it. At the microscopic scale, several studies have demonstrated that the efficiency of the laser coupling with the nano-plasma was enhanced when the electron density approached the critical density.^46,47^ Both effects contribute to the observation of very high levels of laser absorption (up to ∼95%^48^). As a result of the specific interaction dynamics, an optimal laser pulse duration depending on the cluster size has also been observed both for laser absorption^49^ and x-ray conversion.^50^

**FIG. 7.**
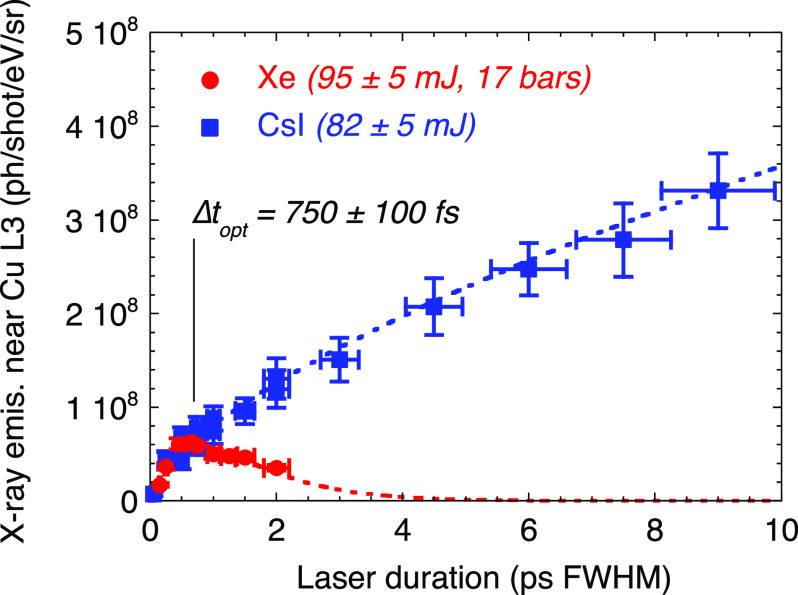
Evolution of the x-ray emission near the L3-edge of copper with the laser pulse duration on target, from a CsI solid target (squares) and from a Xe cluster jet (circles). Reproduced with permission from Dorchies *et al.*, Phys. Rev. E **98**, 033212 (2018). Copyright 2018 American Physical Society.^36^

Figure 7 shows x-ray emission measurements carried out on CsI solid target and xenon cluster jet, with the same setup and near the copper L3-edge. The laser pulse duration has been varied from 30 fs up to ∼10 ps. It confirms the specificity of the laser–cluster interaction. From a practical point of view, the x-ray emission is optimized with a few hundreds of fs laser pulse duration. In principle, it could be increased by using higher pressure (thus larger clusters), but we observed a saturation of the x-ray emission, probably due to the x-ray re-absorption in the surrounding xenon gas.

### B. A shorter x-ray emission

**FIG. 8.**
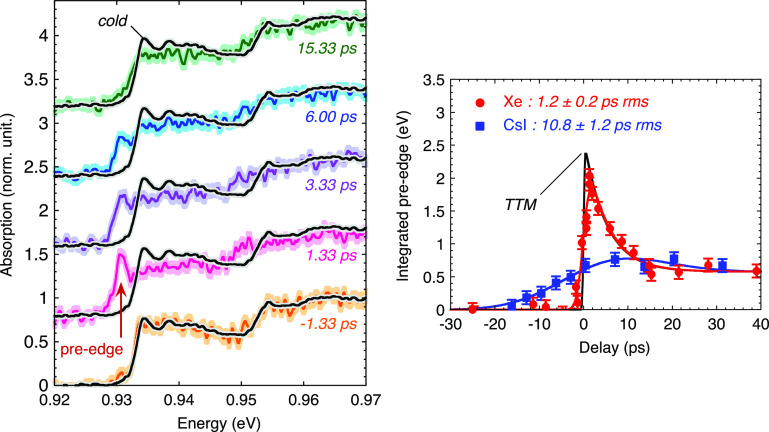
Left: some L3-edge Tr-XANES spectra measured through 80 nm thick copper samples, heated by a fs laser pulse at an incident fluence of 1.4 ± 0.3 J/cm^2^. Each spectrum results from the accumulation of 300 laser shots of 80 mJ, 600 fs on a Xe cluster jet. Reproduced with permission from Jourdain *et al.*, Phys. Rev. B **97**, 075148 (2018). Copyright 2018 American Physical Society.^52^ Right: respective evolution of the pre-edge when probing the sample with the x-ray emission from CsI solid target and Xe cluster jet. The indicated temporal resolution is deduced from the rise time. Reproduced with permission from Dorchies *et al.*, Phys. Rev. E **98**, 033212 (2018). Copyright 2018 American Physical Society.^36^

Another specificity of the laser cluster interaction is that the x-ray pulse duration is expected to be shorter than from solid target. Sub-picosecond duration had been reported for the K-shell emission near 3 keV, in argon clusters.^51^ In order to get similar data for the M-shell emission of a xenon cluster jet, we performed Tr-XANES measurements in femtosecond heated copper foils. Some spectra are presented in Fig. 8 (left). In the same way as with CsI solid target (see Fig. 5), the pre-edge appears just after the laser heating. The increase is here much faster, as it is reported in Fig. 8 (right). These measurements demonstrated a time resolution improved down to 1.2 ± 0.2 ps rms. That is one order of magnitude shorter than with the CsI solid target.

This result was interpreted by a geometrical effect. While a solid target provides a near-critical density area for a long time, where the laser energy can be efficiently deposited, a nanometer expanding cluster very efficiently absorbs the laser energy when the critical density is crossed. Then it quickly turns into underdense plasma where the absorption is drastically reduced. In close correlation, the spherical hydrodynamic expansion of the cluster accelerates the drop in density and temperature, which drastically shortens the x-ray emission. This interpretation was corroborated with numerical simulations, that suggest the x-ray emission duration could reach about 250 femtosecond rms in Xe clusters.^36^ Geometrical considerations contribute to the longer time resolution observed, particularly the close to counter-propagating geometry between the incoming laser and the x-ray collection axis (see Fig. 6).

### C. Selection of some ps Tr-XANES measurements on warm dense copper

Such a short time resolution has been used to investigate the ultrafast dynamics of the transition from solid to warm dense copper. This has already been the subject of several articles^52,53^ that is not the purpose to reproduce extensively in this paper. Let us only mention that the time-resolved pre-edge gave access to the dynamics of the electron temperature, in the first picoseconds following the laser heating. The observations were found in relative good agreement with previous published data on synchrotron,^13,54^ and fairly reproduced by simple Two-Temperature Model (TTM) hydrodynamic calculations (corresponding to the black curve in Fig. 8 (right)). The progressive decrease in *T_e_* is related to the electron-ion thermal equilibration that is well reproduced provided that temperature-dependent parameters are considered, and hydrodynamic expansion is taken into account (more detail in Ref. 52).

**FIG. 9.**
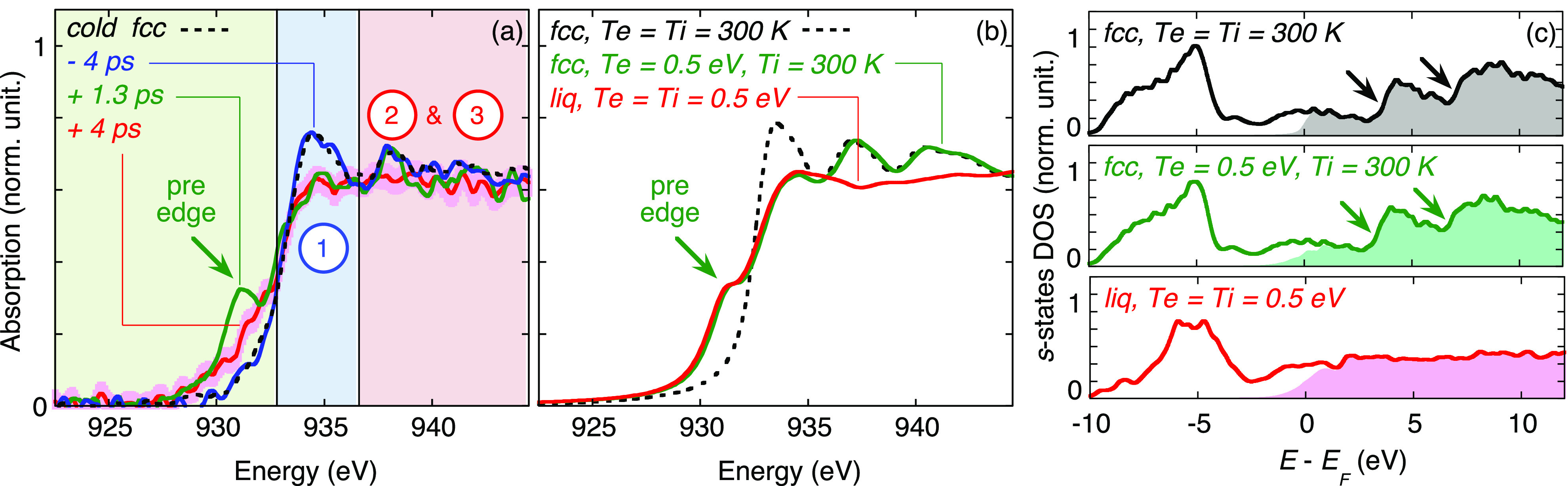
(a) Some Tr-XANES spectra measured at an absorbed fluence of 0.065 ± 0.015 J/cm^2^. (b) Some calculated XANES spectra. (c) Corresponding computed projected DOS on s− states. The van Hove singularities characteristic of the fcc crystalline phase are indicated with arrows. They result in the post-edge peaks labeled 2 and 3 in (a). Reproduced with permission from Jourdain *et al.,* Phys. Rev. Lett. **126**, 065001 (2021). Copyright 2018 American Physical Society.^53^

Another pattern was exploited in the copper L3-edge XANES spectrum. It is illustrated in Fig. 9. A few eV above the edge, small amplitude post-edge modulations were observed without any laser heating (labeled 2 and 3). They were reproduced by DFT calculations, and result from the van Hove singularities in the electron Density Of State (DOS), which are characteristic of the periodic crystalline phase. When the solid turns to liquid, these modulations disappear, signing the melting from the point of view of the electron structure. As such features are more subtle, a higher signal-to-noise ratio was mandatory, requiring accumulation over ∼2000 shots for a single XANES spectrum. A delay was resolved between the rise of the pre-edge (fast increase in *T_e_*) and the post-edge modulations vanishing (loss of lattice periodicity). The characteristic time was observed in the picosecond or even sub-picosecond timescale.^53^ The overall experimental data were well reproduced with two-temperature hydrodynamic simulations, supporting a thermal phase transition.

The interpretation of XANES spectra is of course highly material dependent, and a calculation support is particularly required in the warm dense matter regime where exotic and original situations are expected. However, the investigation carried out on warm dense copper could be *a priori* generalized to other elements. For example, the van Hove singularities are general features in most of the periodic crystalline phases. As a consequence, corresponding post-edge modulations are expected in a wide range of materials. In the same way, the effect of the electron temperature (*T_e_*) on the frontier between occupied and vacant electron states, should be observable on a wide range of x-ray absorption edges, as far as the *T_e_* value exceeds the natural bandwidth of the edge.

Exotic physics is expected in nonequilibrium warm dense matter. For example, bond hardening has been predicted in full d−band metals,^11^ but at $T_{e}\geq 3$ eV. In such a situation, the thermal melting in warm dense copper is expected to occur in the 100 fs scale.^53^ The investigation of this mechanism therefore requires femtosecond resolution, below what can be expected from laser–cluster x-ray source. In addition, the opportunistic sub-ps x-ray source developed from xenon cluster near the copper L3-edge is difficult to generalize to other spectral ranges. Indeed, very few high Z materials exist in gas phase to produce nanometric clusters. An alternative would be to first vaporize a metallic target, but the device would be excessively complex. The future of femtosecond XANES requires other x-ray sources providing both broadband spectrum and ultrashort duration.

## IV. FEMTOSECOND XANES WITH BETATRON X-RAY SOURCE

Betatron radiation from relativistic laser–plasma interaction combines *a priori* ideal features for femtosecond Tr-XANES. Such a table-top x-ray source provides femtosecond pulses (x-ray probe) with a broadband spectrum and inherent synchronization with an optical femtosecond laser pulse (pump). In this section, we present the principle of the betatron x-ray source and its characteristics before to report some fs Tr-XANES experiments performed on nonequilibrium warm dense copper.

### A. Principle of the betatron x-ray source

In synchrotrons, x-rays are emitted from electrons accelerated to relativistic energies in radio frequency cavities and wiggled in periodic magnetic structures (undulators and/or wigglers). All the features of the produced x-ray radiation depend on the electrons orbits, their energy, their amplitude, and period of oscillation.^55^ Betatron x-ray sources reproduce the principle of synchrotrons in a millimeter scale plasma. Electrons are simultaneously accelerated and wiggled in the wake of an intense laser pulse propagating in a low *Z* (atomic number) gas.^56,57^

The idea of accelerating electrons to relativistic energies using laser–plasma interaction was first proposed by Tajima and Dawson in 1979.^58^ Since then, advents in laser technology and in the knowledge of relativistic laser–plasma interaction has led to tremendous progresses in this field of research. Electron sources based on laser–plasma interaction have been developed worldwide. They can now reach the GeV range with a beam quality and reliability always increasing.^59–61^

**FIG. 10.**
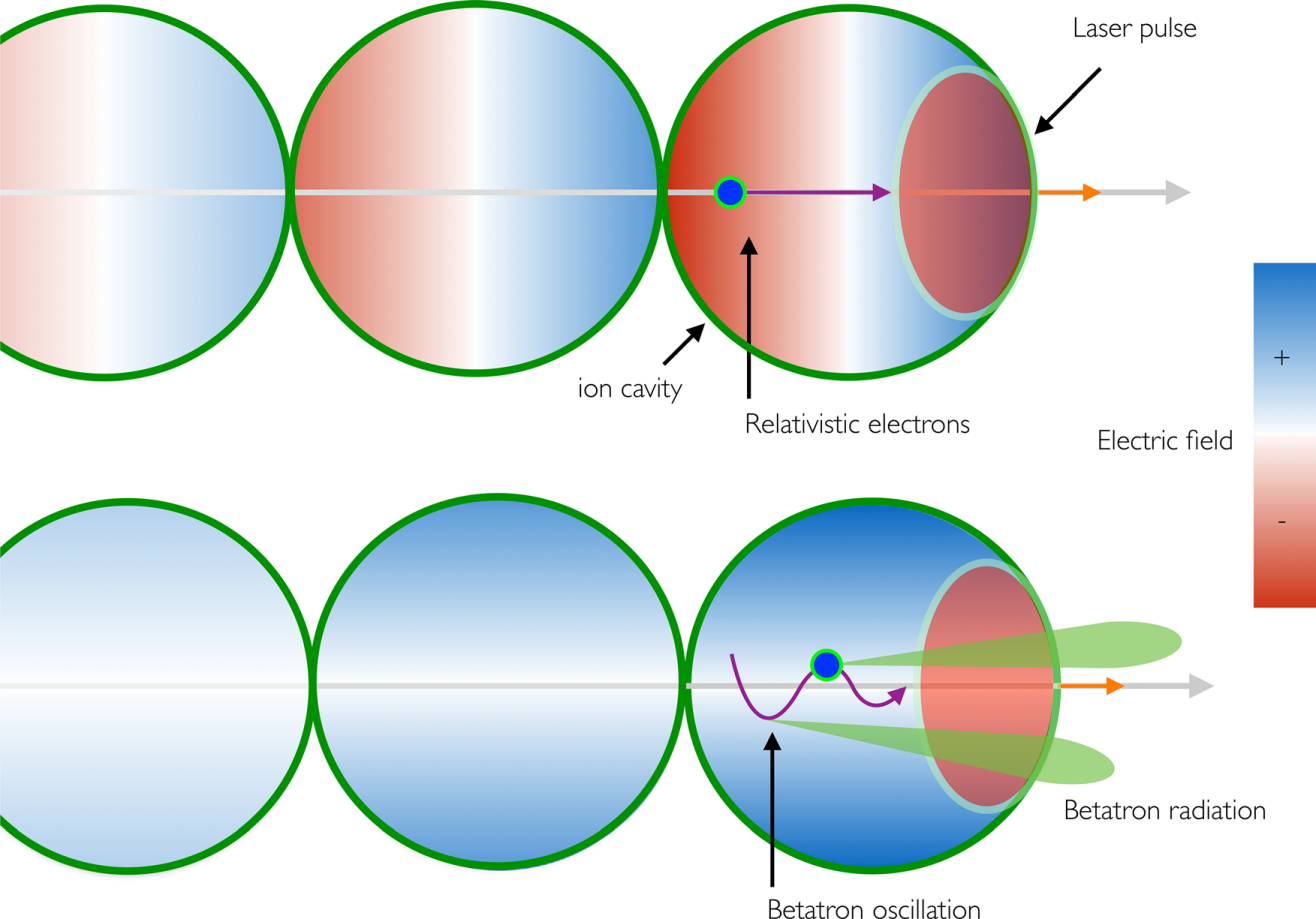
Principle of the betatron source. Top: electrons are accelerated by the longitudinal electric field of the cavity. The electron velocity being larger than the group velocity of the laser, electrons may reach the first half of the cavity and be decelerated. Bottom: the radial electric field is always focusing, and electrons oscillate across the laser axis. Betatron radiation is emitted in the direction of the electron velocity.

The principle of recent laser plasma accelerators (LPA)^62^ is presented in the upper part of Fig. 10. When an intense femtosecond laser pulse propagates in a gas, the pedestal of the laser pulse turns the gas into a plasma. Electrons are first pushed away from high laser intensity regions while ions, much heavier, can be considered as motionless. When electrons are pulled back toward the laser axis, a plasma wave is formed. It propagates in the wake of the laser pulse with a phase velocity equal to the group velocity of the laser. The first arch of the plasma wave is a cavity in which longitudinal and transverse electric fields reach a few hundreds of GeV/m (see Ref. 63). Over the past two decades, several methods have been developed to inject electrons into this cavity.^64–69^ Depending on the method chosen, relativistic electrons beams can be routinely produced with either narrow or broadband energy spectra. For all cases, the duration of the electron bunch is a few femtoseconds only.^70^

In LPA, electrons are both accelerated and simultaneously wiggled. Indeed, electrons are unlikely injected exactly on the laser axis. Most of them are injected off axis and they experience the transverse field of the ion cavity. This field is always focusing, and electrons oscillate across the cavity axis. Their oscillation motion, which is called the betatron oscillation, depends on their transverse position at the moment of injection $r_{\beta }$, their energy (Lorentz factor *γ*), and the plasma density *n_e_*.^71–74^ It is given by

$$\lambda_{u}(\mu m)=4.72\times 10^{10}\sqrt{\gamma /n_{e}({\text{cm}}^{-3})}.$$ (1)

Relativistic electrons undergoing transverse oscillations emit radiation.^57^ This is the so-called betatron radiation (see bottom part of Fig. 10). It is useful to define the parameter *K* (commonly used at synchrotron) to describe the features of the betatron radiation. It is given by

$$K=1.33{\;}^{-10}\sqrt{\gamma \cdot n_{e}({\text{cm}}^{-3})}\cdot r_{\beta }(\mu m).$$ (2)

The radiation is emitted along the direction of the electron velocity. The divergence is given by

θ(rad)=K/γ. (3)

The spectrum is always broadband because the electron motion is highly nonlinear. It can be fitted with a synchrotron function, characterized by a characteristic critical energy *E_c_* given by

$$E_{c}(\text{eV})=5.24\times 10^{-21}\gamma^{2}\cdot n_{e}({\text{cm}}^{-3})\cdot r_{\beta }(\mu m).$$ (4)

After this energy, the photon flux decreases exponentially. The number of photons emitted by one electron along one oscillation period (assuming they all have the mean photon energy $E=0.3\times E_{c}$) is given by

$$N_{\gamma }=3.31\times 10^{-2}\;K.$$ (5)

Finally, the duration is of the same order as the duration of the electron bunch,^75^ that is, a few femtoseconds. From the above-mentioned expressions, an estimation of the radiation properties for constant *λ_u_*, *K*, and *γ* can be obtained for a typical parameter regime (i.e., 50 TW laser focused at intensity in the 10^18^ W/cm^2^ range into a few millimeter gas jet). A 100 MeV electron ( γ∼200) undergoing betatron oscillations in a plasma of density $n_{e}=2\times 10^{19}\;{\text{cm}}^{-3}$ is considered. The spatial period is $\lambda_{u}≃150\mu m$. For *K* = 10, typical of our experimental conditions, the critical energy of the radiation is 5 keV. For three betatron periods, the total number of photons emitted per electron at the mean energy *E* = 1.5 keV is N∼1. Considering that the number of electrons trapped into the ion cavity is of the order of $10^{8-9}$, the number of x-ray photons expected is in the range $10^{8-9}$ as well. Finally, the betatron emission is collimated within a cone of typical solid angle of 50 mrad × 5 mrad.

### B. x-ray source characteristics

**FIG. 11.**
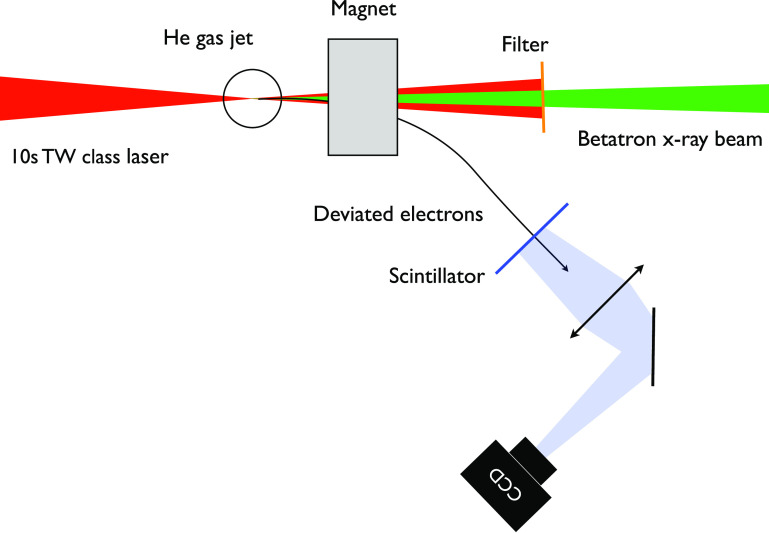
Typical setup of a betatron x-ray source. An intense femtosecond laser pulse (tens of TW) is focused into a millimeter scale gas jet. Relativistic electrons and betatron radiation are produced in the direction of the laser pulse. Electrons are deviated using a magnet. The laser is blocked by a metallic filter that is weakly absorbent for x-rays.

The first demonstration of a betatron source was performed in 2004.^56^ Experimentally, the setup is the same as that for a LPA. As shown in Fig. 11, it simply consists on focusing an intense laser pulse into a gas jet. The parameters that must be appropriately chosen are the focusing optic for the laser (which determine the laser intensity and the Rayleigh length), the density, and the length of the gas target. Typically, the laser intensity must be a few 10^18^ W/cm^2^. The gas length must be a few times the Rayleigh length. So far, the minimum laser power required to produce betatron radiation in the x-ray range (keV range) is a few tens of TW. This type of laser is now widely developed, and compact commercial systems are available.

For the majority of betatron sources, the laser plasma accelerator is used in the regime of ionization injection.^68,76^ In that regime, the gas is helium or hydrogen (99%) with an admixture of nitrogen (1%). The injected electrons originate for the ionization of the nitrogen K-shell and are released within the cavity. It has been demonstrated that this method allows for the production of stable electrons beams.^77^ For that injection mechanism, electrons bunch have a broad continuous spectrum extending up to hundreds of MeV range, with a beam charge of a few hundreds of pC and a divergence of a few mrad. The relativistic electrons are deviated by a magnet placed a few centimeters downstream the gas jet. The x-ray beam can then be characterized, collected with an x-ray optic and used.

**FIG. 12.**
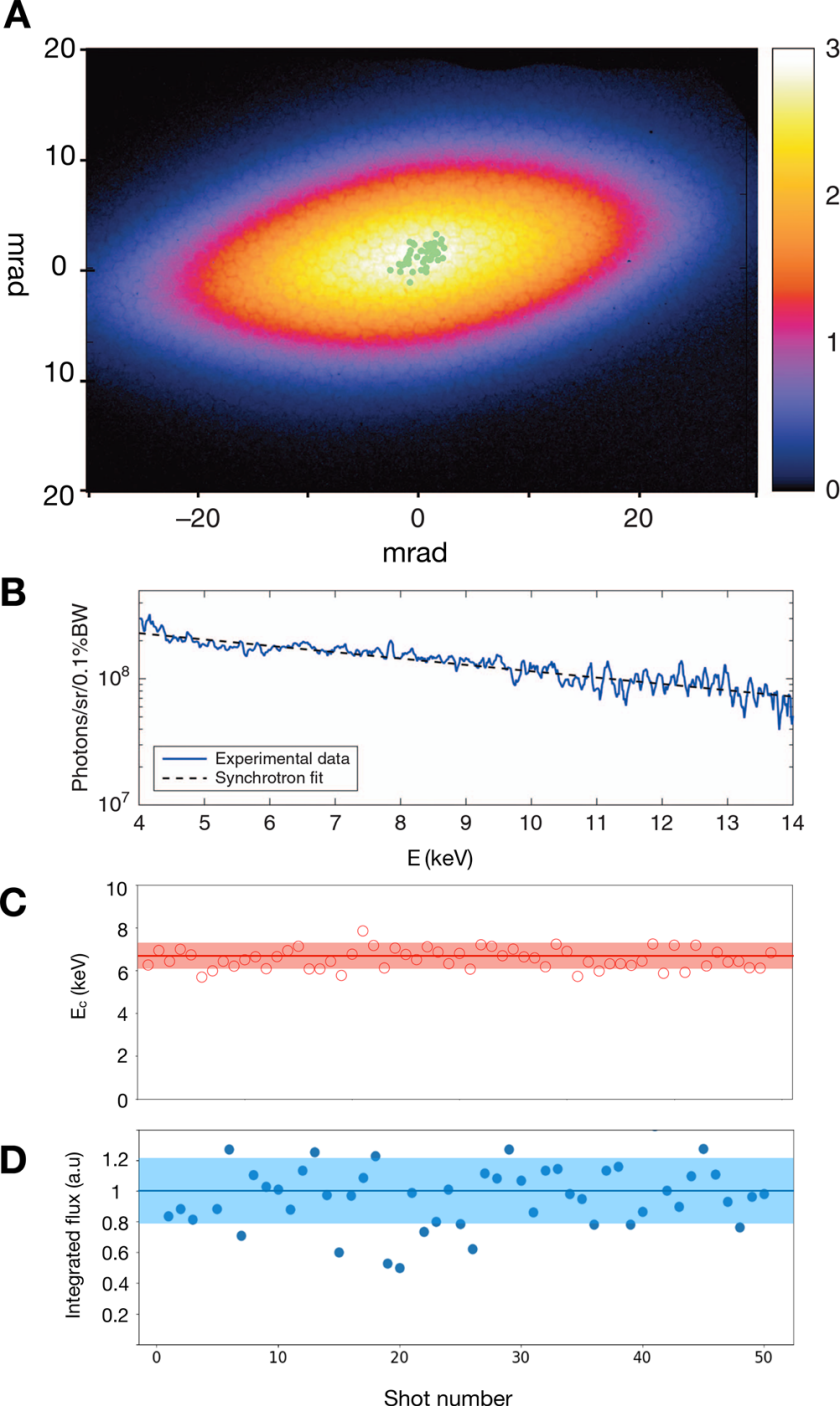
Measurements of the betatron x-ray source in the regime of ionization injection, with a 50 TW laser and a 3 mm long gas jet. (a) Beam profile integrated for consecutive 50 shots. The green dots correspond to the maximum of intensity for each of the shot. (b) Spectrum. (c) Critical energy for 50 consecutive shots. (d) Integrated flux for 50 consecutive shots. Reproduced with permission from Dpp *et al.*, Light **6**, e17086 (2017). Copyright 2017 Springer Nature.^78^

Figure 12(a) presents a typical profile of the x-ray beam. The shape depends on the electrons orbits in the cavity.^78,79^ Here, the elliptical shape is due to the interaction of the electrons with the laser pulse at the moment of injection. Figure 12(b) shows a spectrum obtained under the same conditions. The spectrum can be fitted with a synchrotron function with a critical energy *E_c_* of the order of 10 keV. The photon number is of the order of 10^9^ per shot integrated over the entire spectrum. Figure 12(c) reports the measured stability of the critical energy *E_c_*. The shot to shot stability of the source depends on the stability of the electron beam. At recent experiments, the reported stability is of the order of 10% in energy and flux.

Several methods have been developed to measure the spectrum of the betatron radiation.^80,81^ Spectrometer based on diffraction from crystals or grating, measurement of the x-ray radiation transmitted through metallic filters, or photon counting using an x-ray camera can provide spectra. The choice of the spectrometer is a compromise between the desired resolution and spectral range. Because the spectrum is large, smooth, and continuous, the preferred methods to characterize a betatron source are usually the single photon counting and the measurement of the x-rays through metallic filters.

When higher power lasers are used, such as Petawatt class lasers, the electrons can be accelerated over longer distances and their energy is increased, reaching the GeV range. The betatron radiation they emit becomes more energetic, and the divergence is decreased.^82^ The radiation energy can reach several tens of keV. However, due to the large size and cost of these lasers, they are not, to date, the preferred facilities for applications.

The measurement of the duration of a femtosecond x-ray pulse is not straightforward. For the betatron source, it has been estimated experimentally using pump-probe experiments. The betatron radiation is used to probe an ultrafast phase transition with either x-ray diffraction of x-ray absorption spectroscopy.^28,75^ The resolution of the measurement is limited by the characteristic time of the phase transition. To date, the most accurate measurement obtained shows a pulse duration below 75 fs.^28^ For a more precise result, numerical simulations were performed. The pulse duration obtained using particle-in-cell simulations is ∼10 fs.^28,83^

By combining these numbers, the peak brightness of a betatron source reaches 10^22^ photons per second, mm^2^, and mrad^2^ in 0.1% of the bandwidth (BW). While this peak brightness is greater than in a synchrotron (essentially thanks to the short pulse duration),^71^ betatron sources could work at 10 Hz, but are usually used at a fraction of Hz in practice. This means that these sources should be used in single-shot or several-shots experiments not requiring too much data accumulation.

In addition to the interesting features for Tr-XANES applications (femtosecond pulse duration, broad and smooth continuous spectrum, collimation, and flux), the betatron source has also the advantage of not producing debris (interaction with a gas target). It can be used at the repetition rate of the laser. However, there are two main drawbacks. First, the relativistic electrons produce a background of bremsstrahlung hard x-ray radiation as they go through materials, such as the chamber walls. Lead walls have to be implemented to shield the detectors. Second, the intense laser that drives the source exits the plasma in the same direction as the x-ray beam. It must be blocked with a filter. To avoid significant absorption, a thin filter is required (for 1 keV radiation, we used 400 nm aluminum filter). Such thin filters can easily be damaged by the laser. It must therefore be placed at a position where the laser fluence is acceptable, typically a few tens of centimeters from the source. This implies a minimum distance for the x-ray collection optic that can be placed after the filter. Due to the divergence of the source, the size of the x-ray optic must be sufficiently large to collect most of the beam.

### C. Experimental setup for Tr-XANES measurements

**FIG. 13.**
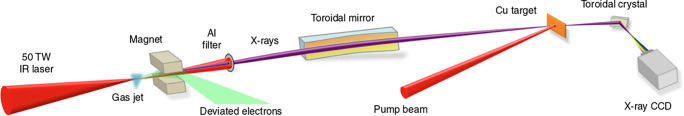
Experimental pump-probe setup designed for femtosecond Tr-XANES studies with a betatron source. The x-rays produced in the gas jet are refocused onto the copper sample. The transmitted spectrum is measured by a Bragg crystal based spectrometer. Reproduced with permission from Mahieu *et al.*, Nature Commun. **9**, 3276 (2018). Copyright 2018 Springer Nature.^28^

In order to take advantage of the work previously carried out at the picosecond timescale,^42,52,53^ we decided to perform the first femtosecond Tr-XANES near the L3-edge of nonequilibrium warm dense copper. As previously mentioned (see Sec. II C), the probed sample and the x-ray detector should be placed far away from the x-ray source, which is the source of parasitic noise. It is even more important with a betatron source: the high pressure gas jet could perturb the sample position between shots, the unconverted laser light needs to be filtered, and the high energy electron beam needs to be removed from the x-ray axis. To overcome all these aspects, we used a mirror at grazing incidence to refocus the x-ray beam on the sample, two meters away from the gas jet. The whole design is presented in Fig. 13, coupled with the betatron source developed in the ionization injection regime described in the last sub-section.

Several aspects were considered to design the x-ray mirror. First, the number of photons was kept as high as possible by adjusting the collection angle close to the betatron solid angle aperture (for this experiment, we measured ∼10 × 20 mrad^2^). Second, the grazing angle was lowered enough to efficiently reflect the x-rays in the spectral range of interest (from 900 to 1000 eV). Third, as mentioned in the last sub-section, the possible damage of the thin foil that filters the unconverted laser was a critical issue, requiring a positioning as far as possible from the gas jet in order to reduce the incident laser intensity. If not designed properly, this filter breaks and the next laser shot could damage all the following optics up to the x-ray CCD. For safety, an automatic monitoring has been set up. The last issue concerned the x-ray focal spot on the sample, which was required as small as possible. Indeed, as it will be further described, the time resolution of the setup strongly depends on it.

To cover all these aspects, we designed a toroidal curved mirror, coated with a 50 nm gold layer. The mirror-source and mirror-sample distances were one meter each. The size of the mirror was 20 × 300 mm^2^, with a grazing angle of 2. The sagittal and tangential radii of curvature were, respectively, 17.5 and 1433 mm, and the reflectivity was about 60%. This geometry gave us enough space to place a strong magnet in order to deflect the electrons in a beam dump. A 100 × 150 *μ*m^2^ FWHM x-ray focal spot was measured on the sample.

The temporal resolution results from several contributions. The x-ray pulse duration is estimated from simulation at $\delta t_{X}=10$ fs. As the x-ray spot size *d_X_* is not negligible, a geometric contribution comes from the angle *α* between the pump and the probe beams (the delay varying along the sample surface). It is estimated by $\delta t_{\text{geom}}=\text{sin}\;\alpha \times d_{X}/c$. We reduced *α* down to 2.2, leading to $\delta t_{\text{geom}}=20$ fs. The experimental uncertainty of the laser beam delay line was estimated to be 1 *μ*m, corresponding to $\delta t_{\text{del}}=7$ fs. A last contribution could be considered, concerning the stability of the sample plane positioning along the x-ray axis. Since the two beams are almost co-propagating, this effect is very small (typically 1 fs for a 500 *μ*m longitudinal shift). However, it could become more critical in a counter propagating geometry (e.g., ∼70 fs for a shift of only 10 *μ*m). The overall temporal resolution is the quadratic sum of these independent contributions: $\delta t=\sqrt{\delta t_{X}^{2}+\delta t_{\text{geom}}^{2}+\delta t_{\text{del}}^{2}}=24$ fs. In order to go below this value, it would be necessary to reduce either the x-ray focal spot, or the angle between the two beams.

The spectrometer needs to respect several constraints: dynamic data acquisition, good spectral resolution, and sensitivity. The main XANES features previously observed in warm dense copper near the L3-edge require a spectral resolution better than 2 eV. The shot-to-shot repetition rate was in practice about 0.2 Hz, limited by the pumping capacity of the vacuum chambers. This was compatible with the use of an x-ray CCD camera with 20 *μ*m pixels. A reflective Bragg crystal was used for x-ray spectral dispersion. Alternatively, a grating could have been used, but its low dispersion would have required a long distance to the CCD (about 10 or 20 m). The drawback is the low reflectivity of crystals.

In order to optimize the number of photons detected per pixel, we designed a spectrometer in Johann's geometry^84^ (curvature along the tangential axis for spectral dispersion), coupled with a curvature along the sagittal axis (for spatial focusing). Another advantage of the Johann's geometry is the non-influence of the x-ray spot size on the spectral resolution. The crystal has been designed in order to collect all the x-ray beam solid angle, while being located far enough from the sample to leave space for the alignment stages. The technical point here was to carefully choose the angles of curvature to match the sagittal focusing plane with the Rowland circle. It ensured the best number of photons per pixel while maintaining the best achievable spectral resolution. Considering all these aspects, we finally chose a RbAP crystal ( 2d=2.612 Å), with a sagittal curvature of 85 mm and a tangential curvature of 200 mm. The estimated integrated reflectivity was 10 mrad.^85^ The center of the crystal was set at 568 mm from the sample and at 100 mm to the image on the CCD.

### D. Selection of some fs Tr-XANES measurements on warm dense copper

The setup described earlier has been used to perform Tr-XANES with a femtosecond time resolution. We followed a data acquisition protocol similar to that used on previous setups: a first set of ∼100 shots to register a reference spectrum (without sample), then a second one to record a cold XANES spectrum through the sample (without the pump laser pulse), and finally a last one through the same but heated sample, at a given delay with respect to the pump. To remove remaining hot spots (hard x-ray noise on the CCD), a median filter was performed in the three dimensions of the stack of hundred images. The signal-to-noise ratio was thus reduced down to the limit of the photon counting statistics.

Despite the definite advantages of the betatron x-ray source (femtosecond pulse with broadband spectrum in a collimated beam), the conversion efficiency from the laser energy ( ∼1 J) to x-rays was still low compared to the thermal laser–plasma sources presented in the Secs. II and III (with only 100 mJ laser). This resulted in a slightly lower number of photons achieved on the sample ( $∼2\times 10^{4}$ instead of $4\times 10^{4}$ ph/eV/shot), then detected by the CCD ( ∼50 ph/eV/shot) near the copper L3-edge.

A first proof-of-principle experiment has been carried out in order to demonstrate the possibility to achieve femtosecond Tr-XANES with betatron x-ray source. A 70 ± 10 nm thick copper foil was heated at ∼1 J/cm^2^. The corresponding spectra are not reported here, but a pre-edge (similar to that previously presented in Fig. 8 for example) was observed to increase very fast with respect to the pump-and-probe delay. The measured rise time demonstrated a time resolution better than 75 ± 25 fs rms.^28^

Such femtosecond time resolution allowed us to investigate new mechanisms of the nonequilibrium warm dense matter physics. Remembering that the electronic temperature *T_e_* can be retrieved from the pre-edge analysis (see Sec. II D), we investigated the electronic transport dynamics in the depth of the sample. Most femtosecond laser experimental studies assume a ballistic transport to justify the ultrafast isochoric production of a homogeneous WDM sample over a thickness of the order of the electron mean free path ( ∼70 nm in copper).^86^ Beyond a critical flux (estimated at $1.5\times 10^{13}$ W/cm^2^ in copper), Chen and co-workers suggested that the ballistic transport is saturated, and the rest of the energy follows a diffusive transport.^87^

**FIG. 14.**
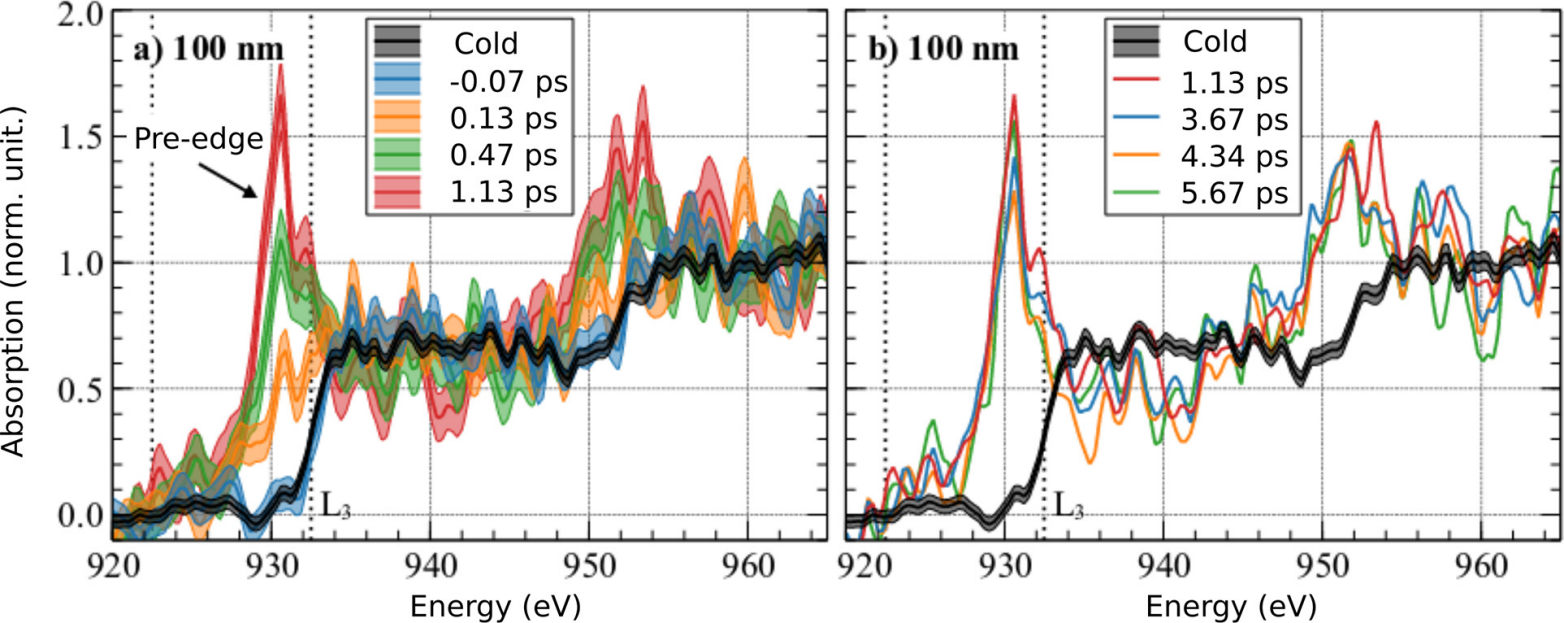
XANES spectra measured at different pump and probe delays, before (a) and after (b) the first picosecond. The sample is a 100 nm thick copper foil heated with an incident fluence of 3.3 ± 0.9 J/cm^2^. The black curve corresponds to the cold XANES spectrum (registered at room temperature).

In Fig. 14, we report some Tr-XANES spectra registered through a femtosecond heated 100 *μ*m thick copper foil. The absorbed flux of $2.5\pm 0.7\times 10^{13}$ W/cm^2^ is about twice the critical value. The photon counting statistics is reported in the error bars (shaded areas). The pre-edge is clearly visible and reaches a maximum level around 1 ps.

**FIG. 15.**
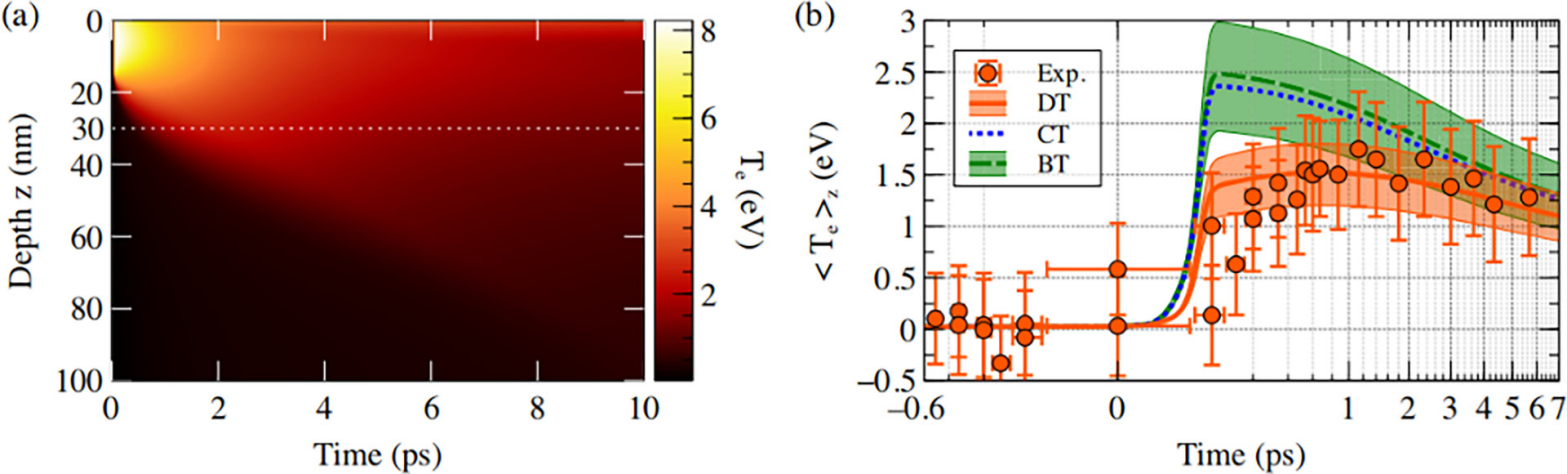
(a) TTM simulation in the case of diffusive transport. It presents the evolution of the electronic temperature *T_e_* as function of depth and time. (b) Comparison of the *T_e_* dynamics with three different TTM simulations: diffusive transport (DT), ballistic transport (BT), and a combination of both (CT). Details are given in the text. Reproduced with permission from Grolleau *et al.*, Phys. Rev. Lett. **127**, 275901 (2021). Copyright 2021 American Physical Society.^89^

The deduced dynamics of *T_e_* was then compared with three different simulations using the two-temperature model implemented in the hydrodynamic code ESTHER.^88^ The first one (BT) assumed a uniformed heating over the whole sample to simulate a ballistic transport. Another one (DT) considered the heating in the laser skin depth only, followed by a diffusive transport in the depth of the sample. The last one (CT) was a combination to simulate the saturation of the ballistic transport. More detail is given in the reference.^89^ An example of a DT simulation is presented in Fig. 15(a). The average electronic temperature was then compared with the experimental results in Fig. 15(b), evidencing that the diffusive transport was the one that best reproduced the data. This femtosecond Tr-XANES has thus enabled to constraint the nature of electron–electron collisions in nonequilibrium warm dense copper.

## V. CONCLUSION

In this paper, we gave an overview of the work we have done for several years on Tr-XANES with ultrafast x-ray sources based on the laser–plasma interaction. Our motivation was the study of the nonequilibrium warm dense matter dynamics when produced by the femtosecond heating of a solid. This imposes constraints on the x-ray source, the most critical of which being the need to get an exploitable XANES spectrum on a limited number of laser shots (considering the nonreversible nature of the heating). This is partially facilitated by the strong changes expected in the XANES spectra from solid to WDM. Over the years, we have developed different types of ultrafast table-top laser-based x-ray sources in order to improve the time resolution from a few ps down to the femtosecond scale. Each of these sources has its advantages and limitations, which are detailed in the body of this paper.

To quickly summarize, the thermal laser–plasma x-ray sources (with solid target or cluster jet) are bright and can be operated with a moderate energy laser ( ∼100 mJ). On the other hand, the spectral range is limited up to ∼4 keV, and the time resolution down to ∼1 ps. These limitations can be overcome with the betatron source produced at higher energy in a gas jet ( ≥1 J). In parallel with X-FELs, which remain unequaled in terms of photon number, the future lies with this table-top x-ray source. It offers both femtosecond resolution and broad spectrum up to over 10 keV. A few years after being discovered,^56^ the betatron source has recently demonstrated its potential for application to femtosecond Tr-XANES,^28^ to the point of allowing realistic physics experiments.^89^ The current limiting point is the still relatively low number of photons available on the sample. However, the improvement of such x-ray source based on laser electron acceleration is far from having reached saturation. Ways exist to significantly increase the level of x-ray emission, by working with more intense lasers,^29^ or even by improving the electron injection and acceleration.^61^

## Data Availability

The data that support the findings of this study are available from the corresponding author upon reasonable request.
